# Understanding Perceptions of the Postsimulation Debriefing Learning Environment in Paediatric Trainees

**DOI:** 10.1111/tct.70203

**Published:** 2025-09-14

**Authors:** Sarah Hoolahan, Anthony Breitbach

**Affiliations:** ^1^ University of Warwick Coventry UK; ^2^ National Maternity Hospital Dublin Ireland; ^3^ Saint Louis University Saint Louis Missouri USA

**Keywords:** paediatric trainees, perceptions, postsimulation debriefing, psychological safety

## Abstract

**Introduction:**

Simulation within medical education develops knowledge, skills and attitudes without risk of patient harm. Debriefing, a two‐way feedback process between learner and facilitator, consolidates learning through a reflective process. Creating a safe learning environment where learners feel comfortable exposing knowledge gaps is essential. Different learners likely perceive the learning environment in unique ways. Although facilitators may strive to create psychological safety, educators and learners can interpret feedback interactions in different ways. The aim of this research was to provide insight into how trainees perceived the postsimulation learning environment.

**Methods:**

Purposeful, comparison‐focused sampling was utilised to recruit participants with varying self‐reported levels of ability, according to the Dreyfus model of skill acquisition, and contrasting perspectives of the debrief. Following random stratification of anonymised survey responses, eight learners participated in semi‐structured interviews. Interviews were transcribed and inductively analysed following a six‐step reflexive thematic analysis process.

**Results:**

Four themes were revealed, which portrayed learner perceptions of the debrief. Feelings and emotions associated with debriefing were both positive and negative, although a fear of judgement prevailed. Safe and unsafe learning environments were described. Feedback was perceived as polite, but not always honest. The impact of both imposter syndrome and cultural norms emerged as unexpected findings. Differences were observed in how those with more experience responded to feedback compared with less experienced colleagues.

**Conclusion:**

Although learners perceived strengths and weaknesses within the postsimulation learning environment, maintaining psychological safety whilst providing honest and credible feedback remains a challenge for educators.

## 1 Background

Simulation within medical education provides an opportunity to practice knowledge, skills and attitudes in a protected environment where inevitable mistakes come without risk of patient harm [1]. Debriefing is defined as a two‐way feedback process between learner and facilitator, typically occurring after the simulation, during which the learner's decisions and actions are explored through a reflective process [2]. This leads to new knowledge formation and consolidation, underpinned by Kolb's experiential learning theory [2, 3]. The debrief is considered the most important aspect of simulation, where the majority of learning outcomes are achieved [1]. Educators perceive a poorly facilitated debrief to be detrimental to learning [4].

*The debrief is considered the most important aspect of simulation, where the majority of learning outcomes are achieved*.

To truly self‐reflect, learners must feel comfortable exposing knowledge gaps and identifying weaknesses [5]. Fear of peer judgement and the perception of a stressful learning environment are considered barriers to debriefing [6]. To overcome this, establishing an environment of psychological safety, defined as feeling secure in taking interpersonal risks, is critical [2, 7].

*Establishing an environment of psychological safety, defined as feeling secure in taking interpersonal risks, is critical*.

Many debriefing models exist to assist facilitators, although there is no evidence to recommend one over another [8]. Previous publications have summarised the common components of debriefing models [3, 8]. It has been acknowledged that psychological safety cannot simply be ‘turned on’, rather it requires facilitators to consciously create the atmosphere they desire [9]. However, there is often incongruence between how educators interpret the purpose and effectiveness of a feedback interaction and how learners perceive the same scenario [8]. Additionally, the hierarchal culture within medicine can further challenge psychological safety, prompting a desire to self‐preserve in the presence of superiors, rather than self‐reflect [10].

Different learners, each with individual needs, will likely perceive the debriefing learning environment in unique ways. Novices, with the least amount of ability, often find simulation daunting in which the level of expected aptitude exceeds their capability [11]. Contrastingly, simulation may burden more experienced learners to demonstrate correct practice at all times, thus limiting their willingness to reveal vulnerability during the reflective process [12].

*Different learners, each with individual needs, will likely perceive the debriefing learning environment in unique ways*.

The aim of this study was to understand how trainees perceive the debriefing environment in simulation, and to explore differing perceptions between novice and more experienced learners.

## 2 Methods

### 2.1 Objectives

The objectives of this study are, firstly, to describe trainees' perceptions of the debriefing environment, secondly, to explore whether perceptions varied depending on the level of experience and, thirdly, to provide guidance for facilitators aspiring to create psychological safety in the debrief.

### 2.2 Study Design and Theoretical Framework

This qualitative study occurred in a tertiary maternity hospital with a well‐established neonatal simulation programme. Postsimulation debriefing conducted as part of this programme was facilitated using the validated TeamGAINS debriefing tool (Appendix 1), which improves psychological safety when implemented by experienced facilitators [13].

This research was shaped by a constructivist paradigm, defined as a belief that multiple truths exist and knowledge is created through the interaction between the researcher and participant to construct each individual's reality [14]. Semistructured interviews were used as they reveal detailed insights into an individual's perspective [15].

### 2.3 Reflexivity Statement

The principal investigator (PI) was an insider researcher, working as a paediatric trainee alongside participants in their clinical role whilst facilitating simulations in their educator role. The attitudes of the researcher inherently influence how data are interpreted and knowledge is created [16]. The PI engaged in regular reflective discourse with their research supervisor to ensure rigorous data collection and analysis whilst remaining sympathetic to the constructivist paradigm and welcoming the influence their own experiences contributed to this research.

### 2.4 Ethical Considerations

Ethical approval was granted by the University of Warwick Research Ethics Committee. A gatekeeper was used during recruitment to ensure voluntary participation. Informed consent was obtained. Data were processed in line with the University of Warwick policies and procedures. Transcripts were anonymised and original recordings destroyed to protect participant anonymity.

### 2.5 Recruitment and Sampling

Purposeful, comparison‐focused sampling was used to identify a predetermined number of participants most likely to provide information‐rich contrasting experiences [17]. This contrasts with thematic saturation, where data are collected until no new themes emerge. Stratified random sampling was used to select from each comparison group to minimise bias. All paediatric non‐consultant hospital doctors (also referred to as trainees) who participated in weekly simulation were invited to participate (*n* = 20).

Participants were recruited via an online survey, issued via the gatekeeper (Appendix 2), which collected demographic information about level of experience in paediatrics and simulation, self‐reported level of ability according to the Dreyfus Five Stage Model of Adult Skill Acquisition (Novice, Advanced Beginner, Competent, Proficient and Expert) [18], and whether the participant found the debrief a positive or negative experience overall, based on Likert scale responses to a series of statements.

Responses were anonymised and subsequently stratified into four subgroups of contrasting characteristics: less experienced learner (self‐reported novice, advanced beginner or competent) with overall positive perception, less experienced with negative perception, more experienced (self‐reported proficient or expert) with positive perception, and more experienced with negative perception. Appendix C1, C2, C3 further describes the recruitment process.

### 2.6 Data Collection

Eight trainees participated in one‐on‐one, face‐to‐face, semistructured interviews, averaging 12 min. An interview guide (Appendix 4) consisted of open‐ended questions to prompt participants, with the aim of collecting rich descriptions [15]. As qualitative research is an iterative process, responses to these initial prompts elicited further individualised questions by the PI to explore unique perceptions in greater detail.

Interviews were conducted by the PI who completed training in qualitative interview techniques as part of their MSc qualification. Participants reflected on any previous debriefing encounters from any training site. Interviews were audio‐recorded, anonymised and manually transcribed verbatim by the PI.

### 2.7 Data Analysis

Reflexive thematic analysis identifies recurring themes across the data set via a six‐step framework as outlined by Braun and Clarke [19]. To answer the research question, theoretical thematic analysis was chosen to capture codes pertaining specifically to trainees' perceptions [19]. Adhering to a peer‐reviewed framework ensured credible analysis of the data whilst remaining sensitive to the subjective role the researcher played in constructing knowledge [20].

## 3 Results

Eight paediatric trainees with differing levels of experience and contrasting perceptions of the debrief participated in this study. The participant characteristics are outlined in Table 1.

**TABLE 1:** Participant characteristics.

Participant 1 (P1)	More experience (ME), negative perception (NP)
Participant 2 (P2)	More experience, positive perception (PP)
Participant 3 (P3)	Less experience (LE), negative perception
Participant 4 (P4)	More experience, negative perception
Participant 5 (P5)	Less experience, positive perception
Participant 6 (P6)	More experience, positive perception
Participant 7 (P7)	Less experience, negative perception
Participant 8 (P8)	Less experience, positive perception

Following an inductive process, four broad themes were discovered: feelings and emotions, the learning environment, feedback and contrasting perceptions.

The first three themes are classified into internal (feelings and emotions) and external (learning environment and feedback) factors that determine how trainees perceive debriefing.

Illustrative quotes for each broad theme are represented in Table 2, whilst subthemes are represented by participant voices within the main text. Table 3 highlights how contrasting perceptions of less and more experienced trainees influence these internal and external factors.

**TABLE 2:** Internal and external factors influencing perceptions.

**Theme 1: Feelings and emotions**	
*Positive Reactions*	*Negative Reactions*
‘Feel good about them afterwards’ *(P1, ME, NP)*	‘I generally dread them’ *(P1)*
‘Relieved that it's over’ *(P1)*	‘Fear of being watched and judged’ *(P1)*
‘Safe’ *(P2, ME, PP)*	‘It was crippling’ *(P1)*
‘Confident’ *(P2 and P5)*	‘I'll squirm’ *(P2)*
‘Comfortable’ *(P3, LE, NP)*	‘Nervous’ *(P3)*
‘I want to come’ *(P5, LE, PP)*	‘Uncomfortable’ *(P4, ME, NP and P8)*
‘I enjoy sims’ *(P6, ME, PP)*	‘Feeling of judgement’ *(P4)*
‘I find them very useful now’ *(P7, LE, NP)*	‘Daunting’ *(P4)*
‘I really loved it’ *(P8, LE, PP)*	‘Intimidating’ *(P4 and P6)*
‘It makes me feel good’ *(P8)*	‘I try to avoid them’ *(P5 and P7)*
‘I feel more confident’ *(P8)*	‘Stressful’ *(P6)*
	‘Embarrassed’ *(P6)*
	‘Anxious’ *(P7)*
	‘Nerve‐wracking’ *(P8)*
**Theme 2: Learning environment**	
*Safe Learning Environment*	*Unsafe Learning Environment*
‘a friendly kind of environment’ *(P5, LE, PP)*	‘I find it a really intimidating environment’ *(P6, ME, PP)*
‘everyone feels they can speak up’ *(P4, ME, NP)*	‘it was kind of finger‐pointing and highlighting people's weaknesses’ *(P7, ME, NP)*
‘I think I felt safe’ *(P8, LE, PP)*	‘there was multiple weeks that NCHDs* would leave sims bawling their eyes out hysterically’ *(P7, ME, NP)* * NCHD = non‐consultant hospital doctor
**Theme 3: Feedback**	
*Challenges with Feedback*	*Desire for Feedback*
‘I've also seen terrible sims where everyone's, em, it was just overly positive in the feedback and I don't think that's beneficial either’ *(P2, ME, PP)*	‘I wouldn't get upset even if someone had kind of negative feedback I think, I think as I said that we probably should be more honest when it comes to feedback like that’ *(P4, ME, NP)*
‘there's an element of honesty that maybe is not always there’ *(P4, ME, NP)*	‘usually there's something to learn and it's useful’ *(P1, ME, NP)*
‘I felt like, em, even when people were giving positive feedback they were only doing that to be nice’ *(P7, LE, NP)*	‘whatever they did wrong, obviously could easily have been me so, yeh, so I think its great because I'll learn from whatever is being commented on for the other person’ *(P2, ME, PP)*
‘I think the feedback is generally more positive from your peers and a bit more, ehm, maybe honest from the facilitators’ *(P6, ME, PP)*	‘I prefer to get feedback rather than keep going and not knowing like which direction or where you can improve on’ *(P8, LE, PP)*
‘feedback to other people, I'm terrible at that, I really don't like it’ *(P1, ME, NP)*	

**TABLE 3:** Contrasting Perceptions.

Feelings and emotions	Learning environment	Feedback
‘If I think back to like when I was a medical student it was crippling, when I think back to intern, SHO* it was challenging, and now I can get past it pretty easily’ *(P1, ME, NP)* *SHO = senior house officer	‘Generally speaking you were safe as an SHO … I was confident enough that I knew the protocol and you got away with not doing too much wrong’ *(P2, ME, PP)*	‘I think because I have quite a good amount of experience I'm comfortable with it and, like I say, I want to do things the right way, I'm here to learn so I'm fine’ *(P3, LE, NP)*
‘I think when you have less experience I feel more pressure, just you don't know what to do and you don't know what's the right thing to do’ *(P3, LE, NP)*	‘more so the more senior you get because you feel like mistakes are just, eh, under a magnifying glass a little bit’ *(P6, ME, PP)*	‘junior person will not feel good from the senior person, if senior person is giving feedback’ *(P5, LE, PP)*
‘when you're a reg* you sometimes have people more junior than you watching on and you feel, or well I feel, nearly embarrassed if I make mistakes in front of more junior staff’ *(P6, ME, PP)* * reg = registrar		

### 3.1 Theme 1: Feelings and Emotions

The contrasting language paints a vivid picture of the divergent reactions trainees have towards the debriefing experience. Whilst some participants did appear to experience more negative sentiments, positive reactions were also captured.

#### 3.1.1 Subtheme 1: Fear of Judgement

The ‘fear of being watched and being judged’ *(P1, ME, NP)* was a strong subtheme, although participants noted this was based on their own insecurities rather than concrete experiences of criticism.

That feeling of judgement that I don't think anyone else has for anyone else but its always just self‐imposed I guess. *(P4, ME, NP)*

Psychological safety strives to remove that fear of judgement by acknowledging that mistakes are inevitable. Despite this, trainees fear others witnessing their shortcomings.

But it's weird, I find it more stressful to be dealing with a doll in front of 10 peers than with a dead baby in front of no peers (laughs), it's, it's tricky. *(P6, ME, PP)*

### 3.2 Theme 2: Description of Learning Environment

Similar to Theme 1, trainees provided contrasting perceptions of the learning environment, with feelings of psychological safety, such as feeling comfortable speaking up and experiencing a welcoming learning environment being described. However, trainees also vividly recalled times their psychological safety was threatened.

#### 3.2.1 Subtheme 2: Co‐Learners Threatened Psychological Safety

Importantly, it was not always the facilitator that created the unsafe environment. One participant recalled an experience where a co‐learner challenged their psychological safety by making them feel like their performance was inadequate.

… leading on and trying to kind of dominate the situation, so I felt uncomfortable. *(P8, LE, PP)*

Another felt that contrasting learning styles could create tension.

I'm very open and straightforward, so when I was giving feedback to the others there was a consultant who I was giving feedback [to], and she, at the end, stopped talking to me … I stopped giving feedback. *(P5, LE, PP)*

The person receiving feedback felt threatened by the content or style in which it was delivered. Interestingly, the interaction resulted in an unsafe learning environment for both learners, as the person giving feedback felt uncomfortable with their colleague's reaction.

### 3.3 Theme 3: Feedback

Trainees perceived feedback as broadly positive, with few experiencing criticism during the debrief. On the contrary, many shared the view that feedback did not address knowledge gaps or correct errors for fear of upsetting the recipient. Participants found feedback from the facilitator rather than peers tended to be more candid and accurate, with trainees acknowledging reluctance to provide feedback to colleagues for fear of causing upset. Despite this, participants expressed a desire for more constructive criticism as an opportunity to learn.

#### 3.3.1 Subtheme 3a: Cultural Impact on Feedback

Trainees, all of whom participated in simulations in Ireland, noted the impact of culture on the way feedback is delivered, and felt there was an Irish tendency to politeness at the expense of directness.

I think it's a very Irish thing as well not to be wholly honest all the time *(P4, ME, NP)*

I think in Ireland anyway the, the problem is people, even if you see something terrible happening in a sim, you're like “oh well I'll say something nice”. *(P6, ME, PP)*

#### 3.3.2 Subtheme 3b: Mechanisms to Improve Feedback

Participants were keen to suggest ways in which corrective feedback could be given whilst maintaining a safe learning environment. These included forewarning participants that feedback would be provided, suggesting concrete ways to improve performance, and providing feedback to all involved in the simulation, not just the team leader.

They more accept [sic] if we say ‘this is only a sim, we are going to comment on each other after that’, people will be more open to that. *(P3, LE, NP)*

… a good way of saying a negative feedback, because she would say ‘oh maybe you can try this one next time’ and like, give an alternative, rather than like ridiculing the mistake. *(P8, LE, PP)*

I think it's important that everyone gets a few things pointed out. *(P2, ME, PP)*

### 3.4 Theme 4: Contrasting Perceptions

A specific objective was to explore differences between trainees with less and more experience. Participants provided rich examples of how perceptions change over time.

Within the first theme, emotions and feelings, more experienced learners reflected on how their anxiety during simulation decreased over time; similarly, less experienced learners described how uncertainty and lack of confidence affected them. However, those with more seniority (typically registrars) also reported a heightened fear of judgement when their less experienced peers were observing them.

Exploring the second theme, the learning environment, trainees felt expectations placed on a less experienced participant (typically senior house officers) were diminished, making the learning environment more secure. Trainees acknowledged that errors were more readily noticed in those with more experience, and the presence of junior colleagues observing these mistakes exacerbated feelings of inadequacy.

Finally, addressing Theme 3, feedback, trainees reported feeling more open to receiving corrective feedback as they gained experience in comparison with those less experienced who believed their confidence could more easily be eroded. Table 3 illustrates these contrasting perceptions.

## 4 Discussion

This study sought to understand how trainees perceive the learning environment during the postsimulation debrief. Participants provided examples of both safe and unsafe learning environments. A fear of judgement prevailed, alongside a reluctance to speak up and offer honest feedback, thus suggesting a lack of psychological safety [7].

*A fear of judgement prevailed, alongside a reluctance to speak up and offer honest feedback, thus suggesting a lack of psychological safety*.

The literature often refers to the responsibility the facilitator has in creating psychological safety, hence the proposition of debriefing models to support educators in this endeavour [8]. However, participants also identified the behaviours of co‐learners as a potential threat to psychological safety. This is important, and may not be immediately apparent to facilitators focusing solely on their own role in promoting psychological safety. Kolbe et al. suggest a strategy of ‘naming the dynamic’ followed by reframing useful aspects of the feedback to initiate reflection in situations where learners criticise each other [9]. This may prove helpful to facilitators in diffusing a challenging situation that could threaten psychological safety.

This research provided an interesting insight into how feedback, a core component of debriefing, is perceived. By virtue of the fact that debriefing is a two‐way process, other learners contribute to the feedback provided. However, trainees described feeling uncomfortable providing negative feedback and perceived that feedback received from peers was less likely to be truthful. Thus, the value and impact of facilitator‐led feedback should not be underestimated. Previous research echoes the discomfort both learners and educators feel when providing corrective feedback [21, 22]. Indeed, apprehension of a negative learner reaction has been identified as a barrier to effective feedback [23]. Pre‐briefing, defined as a conversation prior to simulation where expectations are clarified, can prepare learners to receive corrective feedback [24]. Additionally, as explored in Theme 3, corrective feedback can be normalised by providing concrete examples of how learners can improve and giving feedback to all students within the group.

*Trainees described feeling uncomfortable providing negative feedback, and perceived that feedback received from peers was less likely to be truthful*.

Trainees perceived politeness as a barrier to honest feedback, a phenomenon previously described in the literature [25]. Particularly within the Irish context, there is a cultural tendency towards ambiguous communication in an effort to avoid offence [26]. This study reiterates those findings, with participants highlighting the reticence within Irish culture to give honest feedback. Despite this, participants identified a need for more direct feedback. Educators therefore face a dilemma; how to provide constructive feedback that maintains psychological safety whilst conforming to the cultural norms expected by learners. Further research into understanding the impact of culture, and how this interacts both with institutional norms and the learning environment may be beneficial.

*Particularly within the Irish context, there is a cultural tendency towards ambiguous communication in an effort to avoid offence*.

Another possible interpretation of why trainees do not trust feedback could arise from feelings of imposter syndrome, a common occurrence among physicians [27]. Imposter syndrome describes feelings of self‐doubt in a role and not feeling worthy of success or praise received [27]. Learners expressed a self‐imposed judgement of their performance, which they acknowledged might not be echoed by others. For those suffering from imposter syndrome, it is difficult to accept that praise reflects genuine achievement [28]. Additionally, students asked to ‘perform’ in front of an observer, for example during simulation, are more likely to feel like a fraudulent actor in comparison to how they feel in everyday practice [29]. This may contribute to feelings of imposter syndrome during simulation. This was an unanticipated finding, and further exploration of the impact imposter syndrome has on debriefing, and how it can be minimised, is warranted.

*Learners expressed a self‐imposed judgement of their performance, which they acknowledged might not be echoed by others*.

As anticipated, trainees' perceptions of the debrief changed over time. Those with more experience felt greater reluctance to expose mistakes, reflecting that despite having had less ability, less had been expected from them in the past. Contrastingly, those with less experience encountered uncertainty from not knowing as much as others, which created anxiety during the debrief. Being cognisant of various learners' levels of competency and how that could impact their perceptions of the learning environment may assist educators in fostering psychological safety. Previous research has found that maturity impacts a learner's receptivity to feedback [30]. Our findings support this, with participants advocating for negative feedback to be minimised for those less experienced who may be easily discouraged, whilst those with more experience could handle criticism without diminishing their confidence. We encourage facilitators to tailor feedback to the learner's expected level of ability.

*We encourage facilitators to tailor feedback to the learner's expected level of ability*.

### 4.1 Limitations and Potential Bias

In some qualitative studies, data are collected until saturation is reached. Data collection was pre‐determined in this study; however, the intention of purposeful sampling is to ensure saturation is achieved by obtaining rich insights from a contrasting cohort. Given the insider researcher role the PI played, additional stratified random sampling was utilised to reduce bias when selecting participants.

As the PI was also a simulation facilitator, participants may have felt uncomfortable recounting negative experiences from their current simulation programme. This may have limited discourse, leading to an inaccurate portrayal of the learning environment. A gatekeeper and co‐debriefer not involved in the study were utilised to minimise this risk. Additionally, trainees were invited to discuss experiences without specifying when or where they took place.

Trainees were asked to self‐report their level of competency, which may have introduced bias. Descriptive definitions were provided to assist participants in accurately selecting their competency level; however, it is possible trainees under or overestimated this.

Interviews were relatively short; however, no time limit was enforced, and trainees were encouraged to provide as much description as they wished. The rich narratives suggest these interviews were of sufficient duration; however, longer interviews may have garnered more detailed insights.

## 5 Conclusion

This research sought to answer the question of how trainees perceived the debriefing learning environment and identified both internal (feelings and emotions) and external (learning environment and feedback) factors that influenced perceptions. Additionally, we explored how perceptions varied between trainees with less and more experience. Despite a large body of literature surrounding psychological safety, it is evident learners do not always perceive the postsimulation learning environment as safe. This study contributes to this literature by exploring the influence of societal culture on the effectiveness of debriefing and by addressing the role imposter syndrome may play during the debrief. Further research into the interesting dichotomy of providing honest feedback whilst supporting learners to trust the feedback they receive may provide beneficial guidance for educators.

*Despite a large body of literature surrounding psychological safety, it is evident learners do not always perceive the postsimulation learning environment as safe*.

## Author Contributions

**Sarah Hoolahan:** conceptualization (lead), formal analysis (lead), software (lead), writing – original draft (lead), writing – review and editing (equal). **Anthony Breitbach:** writing – review and editing (equal).

## Ethics Statement

Ethical approval was granted by the University of Warwick Biomedical and Scientific Research Ethics Committee. Data were processed in line with the University of Warwick policies and procedures. The first author undertook this research as part of the Master's in Medical Education qualification at the University of Warwick.

## Conflicts of Interest

The authors declare no conflicts of interest.

## Data Availability

The data that support the findings of this study are available from the corresponding author upon reasonable request.

## Appendix 1 TeamGAINS Debriefing Tool

Kolbe, M. et al. (2013). TeamGAINS: a tool for structured debriefings for simulation‐based team trainings. BMJ Quality and Safety, 22 (7), 41–553. Available at: 10.1136/bmjqs-2012-000917 23525093

## Appendix 3 Recruitment Process

An overall positive or negative perception was determined based on responses to five Likert scale statements. Each statement was scored out of 5: *strongly disagree* = 1 point, *disagree* = 2 points, *neutral* = 3 points, *agree* = 4 points, *strongly agree* = 5 points.

These scores were summated for each participant, and a median score was calculated based on all responses. Those who scored below the median were considered to have a relatively negative perception, whilst those who scored above were deemed to have a relatively positive perception. Finally, an online random number generator was used to randomly select two participants from each subgroup who were invited to participate in an interview.

**TABLE C1:** Breakdown of each respondent's characteristics.

Respondent	Dreyfus level of ability	Total Likert score
R1	Proficient	20
R2	Proficient	26
R3	Expert	29
R4	Competent	27
R5	Advanced beginner	20
R6	Competent	25
R7	Proficient	24
R8	Competent	27
R9	Competent	27
R10	Advanced beginner	28
R11	Proficient	26
R12	Competent	19

**TABLE C2:** Final breakdown of respondent characteristics.

	Negative perception (< 26)	Positive perception (≥ 26)
Less experience	R5 R6 = P3 R12 = P7	R4 R8 R9 = P5 R10 = P8
More experience	R1 = P1 R7 = P4	R2 R3 = P2 R11 = P6

*Note:* R = respondent, P = participant; underlined text represents those randomly selected to participate in interview.

**TABLE C3:** Final breakdown of selected participants.

	Negative perception	Positive perception
Less experience	P3	P5
	P7	P8
More experience	P1	P2
	P4	P6

## Appendix 4 Interview Guide

**Table:**

1. Can you tell me how you felt during the debrief?
2. Were there any parts that made you feel uncomfortable? Can you tell me more about that? Why did you think it made you uncomfortable?
3. Was there anything that the facilitators did that made you feel more or less comfortable?
4. How did you feel knowing that other people more experienced/less experienced than you were watching your performance?
5. How comfortable did you feel when the facilitator was giving you feedback?
6. Did you find the feedback helpful or unhelpful? Can you tell me more about that?
7. If you were facilitating the sim, is there anything you would do differently during the debrief?
